# A Low-Noise Hybrid-Integrated Balanced Homodyne Receiver with 2.5 GHz Bandwidth and 15 dB Quantum Shot Noise Clearance

**DOI:** 10.3390/mi16121416

**Published:** 2025-12-17

**Authors:** Yihao Yang, Chao Cheng, Ruixuan Yang, Yangming Ren, Shenlei Bao, Jintao Xue, Houyou Lai, Binhao Wang

**Affiliations:** 1 State Key Laboratory of Ultrafast Optical Science and Technology, Xi’an Institute of Optics and Precision Mechanics, Chinese Academy of Sciences, Xi’an 710119, China; yangyihao211@mails.ucas.ac.cn (Y.Y.); chengchao2022@opt.ac.cn (C.C.); renyangming2017@opt.ac.cn (Y.R.); baoshenlei@opt.ac.cn (S.B.); xuejintao@opt.ac.cn (J.X.); laihouyou24@mails.ucas.ac.cn (H.L.); 2 School of Future Technology, University of Chinese Academy of Sciences, Beijing 100049, China; 3 Faculty of Electronic and Information Engineering, Xi’an Jiaotong University, Xi’an 710049, China; yrxay358@stu.xjtu.edu.cn

**Keywords:** balanced homodyne receiver, optoelectronic co-design, low-noise transimpedance amplifier

## Abstract

The rapid development of continuous-variable quantum communication has driven an increasing demand for high-performance quantum signal processing modules. Among these, the balanced homodyne detector (BHD) has emerged as a leading solution for practical quantum state measurement due to its capability to provide complete quantum mechanical characterization. However, its performance is often constrained by limited bandwidth and high noise levels, primarily due to the reliance on bulk optical components and discrete receiver electronics. The dominant noise source in these systems typically stems from electronic noise, while imbalances in the optical path further degrade the signal-to-noise ratio (SNR) of the BHD. In this work, we present an adjustable integrated optical path to enhance the balance within the BHD system, along with a low-noise transimpedance amplifier (TIA) by employing optoelectronic co-design. Our design achieves a bandwidth of 2.5 GHz, an input-referred noise current of only 2 pA/√Hz in 180 nm CMOS technology, and a measured quantum shot noise clearance of 15 dB generated from a 700 μA photocurrent. This is the maximum quantum shot noise clearance at the same BHD photocurrent reported to date above the GHz bandwidth.

## 1. Introduction

**Figure 1:**
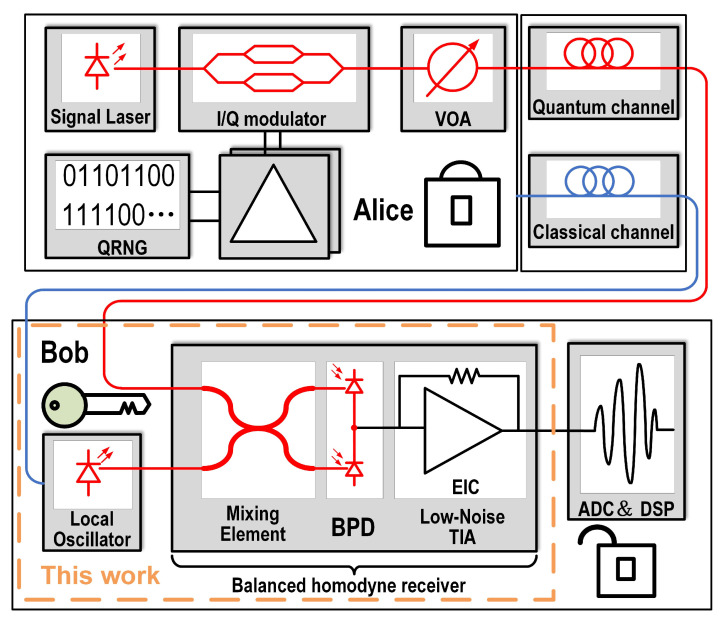
Block diagram of the continuous-variable quantum key distribution system.

High-speed balanced homodyne receivers have become essential building blocks in a multitude of applications that demand high levels of sensitivity [1]. Unlike applications in optical communications, the noise performance of a balanced homodyne receiver is particularly important in quantum measurements due to the requirement for unconditional secrecy [2]. Figure 1 illustrates a typical continuous-variable quantum key distribution (CV-QKD) system. On the transmitter side (Alice) [3], a laser source is used in conjunction with an in-phase and quadrature (IQ) modulator. The transmitted symbols, generated by a quantum random number generator (QRNG), are attenuated using a variable optical attenuator (VOA) to achieve the desired power level. On the receiver side (Bob), the incoming optical signal is combined with a local oscillator (LO) laser via a mixing element and measured using a BHD. To ensure unconditional security, the transmitted optical symbols must operate at the quantum noise limit, as dictated by the Heisenberg uncertainty principle [4,5,6]. This stringent requirement necessitates that the TIA, which converts the photocurrent from the BHD into a voltage signal, exhibits extremely low noise characteristics. Finally, the signal is digitized using an analog-to-digital converter (ADC) for further processing. Therefore, one of the key performance requirements for a balanced homodyne receiver in a CV-QKD system is to minimize electronic noise while maintaining sufficient bandwidth [7]. However, according to [8], the TIA, as the core of the balanced homodyne receiver, inherently faces a trade-off between bandwidth and noise, which would constrain the capture of quantum information.

In this work, we present an integrated balanced homodyne receiver with a comprehensive noise reduction strategy for the TIA. We employ a positive feedback Miller capacitor to boost the bandwidth of the TIA and free up space for maximizing the feedback resistance RF of the TIA, as well as increasing the transconductance of the input transistors. Our design achieves a 15 dB quantum shot noise clearance with an input photocurrent of 700 μA, a bandwidth of 2.5 GHz, and an input-referred noise current of only 2 pA/√Hz in 180 nm CMOS technology, which is significantly better compared to previous designs. Additionally, we introduce an adjustable integrated optical path to enhance the balance within the BHD system, further improving the balanced homodyne receiver’s performance.

## 2. Optical Circuit Design

**Figure 2:**
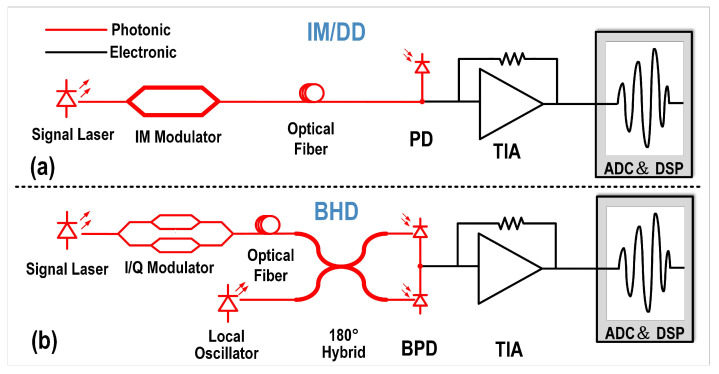
Schematic diagrams of (**a**) IM/DD and (**b**) BHD configurations.

Figure 2a illustrates the intensity modulation direct detection (IM/DD) link. The optical carrier is intensity-modulated (either by direct modulation of the laser or via an external intensity modulator) and then launched into the optical fiber. At the receiver, the optical signal is converted into an electrical current by a photodetector (PD). The PD current is amplified by a TIA and subsequently sampled and quantized by an analog-to-digital converter (ADC). Finally, the digital signal processing (DSP) block performs signal recovery and detection. However, this transmission scheme requires high-sensitivity detectors at the receiver (RX) to detect relatively weak light signals. At the same time, the PD must also provide sufficient bandwidth to capture rapid signal variations.

Balanced homodyne detection employs a local oscillator (LO) that interferes with the incoming optical signal to enable balanced detection, as illustrated in Figure 2b. This detection scheme provides access to orthogonal optical field quadrature without requiring ultrafast detectors. Moreover, the LO effectively amplifies the detected optical power. When a strong LO is used, the beat signal can be significantly enhanced, thereby improving the overall detection sensitivity. As a result, the output signal can rise above the electronic noise floor, allowing extremely weak optical signals to be accurately measured. Table 1 summarizes and compares the characteristics and requirements of IM/DD and BHD schemes. Although BHD imposes lower bandwidth and sensitivity requirements compared to IM/DD, it entails higher power consumption and greater design complexity. Therefore, the optimal solution for RX is to use a single-ended TIA to receive the difference between the two PD output optical currents, then convert the single-ended signal to differential via a circuit before processing by the DSP module. However, the larger parasitic capacitance of the two PDs limits the RX’s bandwidth and noise performance.

Conventional balanced homodyne receivers employ a 50/50 multimode interferometer (MMI) to generate two beams of equal intensity. The input and LO optical signals are denoted as *E_S_* and *E_L_*, respectively, with their expressions given by (1) and (2). Here, $A_{S}$ and $A_{L}$ represent the amplitudes of the optical signals, $\omega_{S}$ and $\omega_{L}$ represent their respective carrier frequencies, while $\varphi_{S}$ and $\varphi_{L}$ denote their respective phases and $\varphi_{N}$ represents the phase noise. Two photodiodes with nearly identical responsivity then convert these beams into photocurrent signals, which are subsequently subtracted. Equations (3)–(6) describe the magnitude of the photocurrent in each PD and the output photocurrent after passing through a 180° mixer, where $P_{S}$ and $P_{L}$ represent the optical power of the input optical signal and the local oscillator light, respectively, K is the proportional coefficient, and $I_{T}$, $I_{B}$, and $I_{O}$ illustrate the photocurrents in the top PD, the bottom PD, and the output photocurrents, respectively. $R_{T}$ and $R_{B}$ denote the responsivities of the two PDs. When the MMI operates ideally and the two PDs have identical responsivities, i.e., $\varphi_{N}$ = 0, $\omega_{S}$ = $\omega_{L}$, $\omega_{d}$ = 0, $R_{T}$ = $R_{B}$, and $\varphi_{S}$ − $\varphi_{L}$ = 0° or 180°, the output current can be expressed as $I_{O}$ = $\pm (R_{T}+R_{B})\sqrt{P_{S}P_{L}}$. This process cancels the classical noise, while the shot noise is converted into a voltage signal by a TIA. However, any asymmetry in the MMI or between the two PDs allows portions of the classical noise and DC components to remain. These residual signals are also amplified by the TIA and forwarded to the information processing module.

(1) $$E_{S}=A_{S}\;\exp\left[-j\left(\omega_{S}t+\varphi_{S}+\varphi_{N}\right)\right]\;$$

(2) $$E_{L}=A_{L}\;\exp\left[-j\left(\omega_{L}t+\varphi_{L}\right)\right]$$

(3) $$\left\{\begin{array}{c} \;P_{S}=KA_{S}^{2}\\ P_{L}=KA_{L}^{2}\\ \omega_{d}=\omega_{S}-\omega_{L} \end{array}\right.$$

(4) $$I_{T}=\frac{1}{2}R_{T}(P_{S}+P_{L}+2\sqrt{P_{S}P_{L}}\;\cos\left(\omega_{d}t+\varphi_{S}+\varphi_{N}-\varphi_{L}\right)$$

(5) $$I_{B}=\frac{1}{2}R_{B}(P_{S}+P_{L}-2\sqrt{P_{S}P_{L}}\;\cos\left(\omega_{d}t+\varphi_{S}+\varphi_{N}-\varphi_{L}\right)$$

(6) $$I_{O}=I_{T}-I_{B}=(R_{T}+R_{B})\sqrt{P_{S}P_{L}}\;\cos\left(\omega_{d}t+\varphi_{S}-\varphi_{L}\right)+\frac{1}{2}(R_{T}-R_{B})\left(P_{S}+P_{L}\right)$$

**Figure 3:**
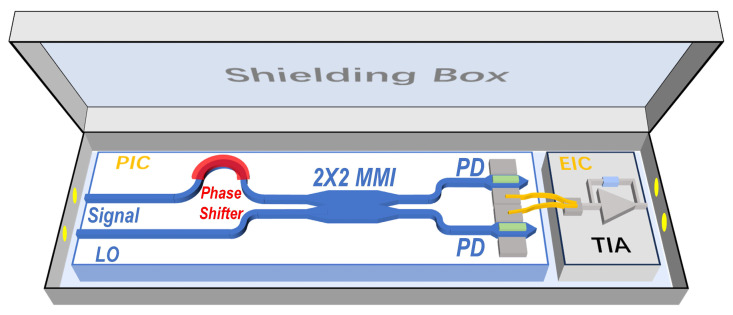
Schematic of the proposed integrated balanced homodyne receiver and the experimental measurement environment.

Figure 3 illustrates the hybrid integration scheme employed in this work. An adjustable phase shifter is positioned between the input port and the 50/50 MMI to modulate the phase of the input optical signal through a thermo-optic effect. According to (4)–(6), adjusting the phase of the input signal can compensate for the phase errors introduced by asymmetries in the MMI and PD, and the term $\varphi_{N}$, thereby achieving accurate demodulation. Simultaneously, during quantum shot noise clearance measurement, the phase shifter is also used to adjust $\varphi_{S}$, thereby controlling the magnitude of the cosine terms in Equations (4) and (5). This correction mitigates the DC offsets in $I_{T}$ and $I_{B}$ caused by inconsistencies between $R_{T}$ and $R_{B}$. Intuitively, manufacturing errors in the MMI and PDs can cause inconsistencies in the photocurrent output between the two PDs. By fine-tuning the input optical phase, the phase shifter adjusts the optical power distribution at the MMI outputs, compensating for optical-path asymmetries and ensuring equal photocurrent magnitudes at both PDs. During the measurement process, the deviation between the two PDs outputs can be directly determined by the DC value at the EIC output node (in balanced state at 1.3 V). Then, the phase shifter is adjusted for fine-tuning by varying the voltage applied to the phase shifter, which requires approximately 20 mW of power to achieve a π-phase shift, ultimately ensuring that the photocurrents from both PDs are equal in magnitude. Subsequently, the output photocurrent signal is fed into the electronic chip via two bonding wires, where an ultra-low noise TIA serves as the core component. During testing, the proposed balanced homodyne receiver is enclosed in a shielding structure to minimize external environmental interference. Fiber and high-speed signal lines pass through designated cut-outs in the shield, which is internally lined with reflective, wave-absorbing material to further reduce noise.

**Figure 4:**
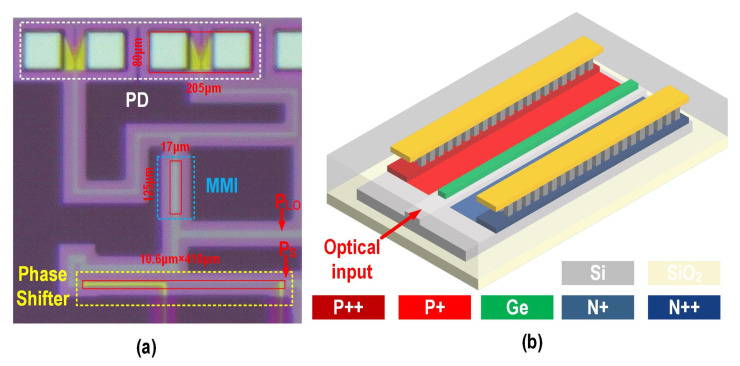
(**a**) Die micrograph of the fabricated BHD and (**b**) three-dimensional schematic of the proposed Si-Ge PD.

Figure 4a shows the die micrograph of the proposed BHD fabricated in a 130 nm SOI platform, which occupies an area of 570 μm × 540 μm. A TiN heater with dimensions of 106 μm × 410 μm is positioned between the input optical signal and the 50:50 2 × 2 MMI (with an insertion loss below 0.3 dB). The heater terminals are connected to metal electrodes that supply the heating voltage, inducing a phase shift in the optical signal within the waveguide through the thermo-optic effect. The output waveguides connecting the 2 × 2 MMI to the two PDs are symmetrically bent to ensure equal optical path lengths. The common terminals of the two PDs are wire bonded to the input pads of the TIA to verify PD consistency before the optoelectronic co-integration measurement. The relatively large spacing between the two bonding wires minimizes mutual inductance. Compared to bonding a single pad with two wires to the TIA input, the parasitic inductance between the photonics integrated circuit (PIC) and electrical integrated circuit (EIC) in the interconnect is significantly reduced. However, this approach also introduces additional parasitic capacitance from the pad to the TIA input, which will be mitigated in the EIC design.

**Figure 5:**
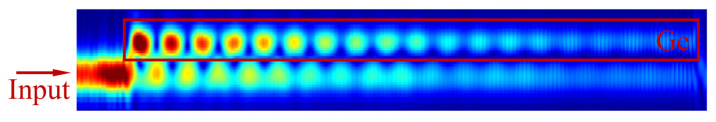
Simulated optical field distribution of the Si-Ge PD.

Figure 4b presents a three-dimensional schematic of the lateral PIN silicon–germanium (Si-Ge) PD. The incident O-band light is coupled into the Ge absorption layer through the evanescent field of the rib waveguide, where it is subsequently absorbed to generate photocurrent. To achieve an optimal trade-off between responsivity and optoelectronic (OE) bandwidth, a relatively narrow Ge absorption region is adopted to effectively shorten the transit time of photogenerated carriers. Meanwhile, to mitigate the potential degradation in responsivity caused by the reduced cross-sectional area, the Ge stripe is designed with an extended length. Considering these design optimizations, the final dimensions of the Ge layer are 30 μm × 0.5 μm × 0.26 μm (length × width × height). In the doping design, heavy doping is introduced near the ohmic contacts to reduce the device’s resistance. Figure 5 illustrates the simulated optical field distribution of the PD. It can be clearly observed that, when the Ge absorption length is 30 μm, the incident light is almost completely absorbed.

## 3. Optoelectronic Co-Design

**Figure 6:**
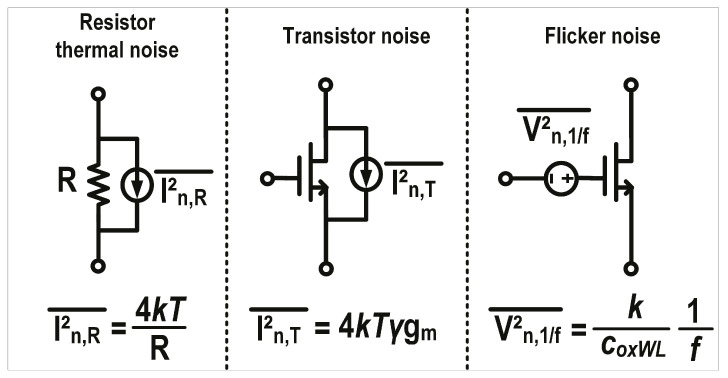
Schematic diagram of noise sources in the EIC.

The noise sources in the EIC primarily consist of three components: resistor thermal noise, transistor noise, and flicker noise. Figure 6 depicts the equivalent schematic of these three noise sources within the electronic circuit. Resistive thermal noise arises from the random motion of electrons within conductors. Its noise spectrum is proportional to absolute temperature (*T*), while the magnitude of its equivalent current noise is inversely proportional to resistance (R). Transistor noise is thermal noise generated by the irregular motion of charge carriers in the transistor’s channel. It is associated with the process-dependent constant *k* and the noise coefficient γ. Its noise spectrum is positively correlated with T and the transistor’s transconductance (g_m_). The primary source of flicker noise is the random trapping of charge carriers by suspension bonds between the gate oxide layer and the silicon substrate. Its noise spectrum is inversely proportional to the transistor’s gate area (*WL*), capacitance of the gate oxide per unit area (*C_OX_*), and frequency (*f*) and is also called 1/*f* noise.

**Figure 7:**
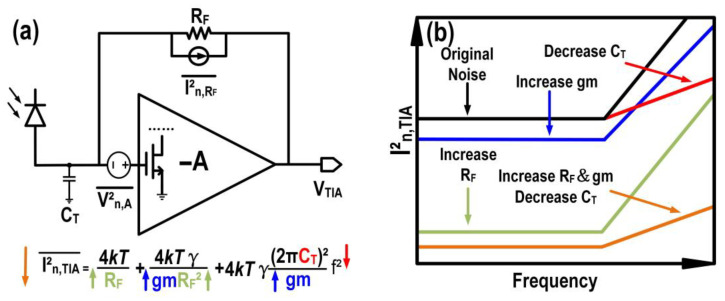
(**a**) SFTIA front-end topology and (**b**) noise analysis.

We begin by reviewing the shunt feedback TIA (SFTIA), shown in Figure 7a, with annotated noise sources to identify the primary factors limiting its noise performance. The equivalent input-referred noise current of an SFTIA is primarily determined by the feedback resistor (R_F_), total input capacitance (C_T_), and the noise contributions of the input transistor. Figure 7b highlights the theoretical input-referred noise current power spectral density and corresponding strategies for its reduction. As reported in [9], increasing R_F_ improves noise performance. Additionally, minimizing parasitic capacitance mismatch between the input transistor and the combined parasitic capacitance of the TIA’s pad and the PD (C_pad_ + C_PD_) is crucial [10]. This is because the g_m_ is directly proportional to its parasitic capacitance, thereby restricting the effectiveness of increasing g_m_ as an approach to reduce the input-referred noise current [11]. Furthermore, decreasing the C_T_ is also necessary as it reduces the noise that is positively correlated with the frequency (f). Overall, optimal performance is achieved by increasing R_F_ and g_m_ while reducing the C_T_.

The 3 dB bandwidth (BW) and the transimpedance limit of the SFTIA can be expressed as follows, assuming a Butterworth response [12]:

(7) $$BW=\sqrt{\frac{Af_{A}}{2\pi C_{T}R_{F}}}$$

(8) $$\;\;\;\;\;\;\;\;\;R_{F}\leq \sqrt{2^{n}+1}\;tan^{n}(\frac{90{}^{\circ}-\emptyset_{m}}{n})\frac{{\left(Af_{A}\right)}^{n}}{2\pi C_{T}BW^{n+1}}$$

Here, $\emptyset_{m}$ represents the phase margin of the TIA loop, while n, A, and $f_{A}$ denote the number of gain stages, the gain, and the 3 dB bandwidth of the core amplifier, respectively. From (7) and (8), it can be inferred that increasing R_F_ reduces the BW of the SFTIA. Conversely, a larger gain–bandwidth product (GBP) of the core amplifier and a smaller total input capacitance C_T_ lead to a higher BW and allow the use of a larger R_F_.

**Figure 8:**
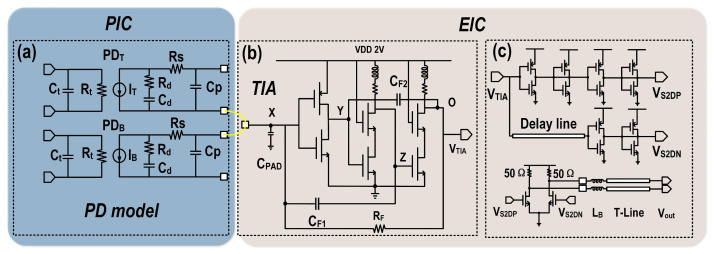
(**a**) Schematic diagram of the co-designed photodetector model, (**b**) proposed TIA, and (**c**) S2D with output buffer.

Figure 8a illustrates the proposed equivalent circuit model of the PD for optoelectronic co-design. The design considerations for a PD in a high-speed and low-noise optical receiver front-end are often multifaceted. Developing an accurate and experimentally validated equivalent circuit model is essential for CMOS circuit co-design and serves as an efficient tool for iterative optimization. As shown in Figure 8a, the PD equivalent circuit model consists of carrier transit time effect and electrical parasitics. The carrier transport dynamics is modeled by an RC (R_t_ and C_t_) input network to describe the motion of photocarriers in Ge and Si [13], and the electrical parasitics include contributions from the absorption region, substrate, and interconnects. The absorption region is modeled with a drift resistor R_d_ and a junction capacitor C_d._ While R_s_ denotes the diode series resistor, C_p_ represents the parasitic capacitance associated with the substrate and contact pads. The parameters in the equivalent circuit model can be extracted by curve fitting the PD’s small signal responses. The PIC and EIC are interconnected using two gold bonding wires, which significantly reduce parasitic inductance but introduce additional parasitic capacitance. This trade-off limits the overall bandwidth of the balanced homodyne receiver and increases the input-referred noise current of the TIA.

Figure 8b illustrates the proposed TIA structure, employing a three-stage inverting amplifier as the core amplifier of the TIA to provide sufficient gain for the feedback loop. In addition to this, the second and third stage amplifiers utilize cascode transistors to isolate the negative feedback Miller effect of each stage [14]. Based on (7) and (8) and Figure 7, this approach allows increasing the RF value while maintaining the same bandwidth, thereby reducing the input-referred noise current of the TIA. However, the junction capacitance of the two PDs in the BHD and the parasitic capacitance of the two pads still limit both bandwidth and noise performance. To address this issue, we employ positive feedback Miller capacitance compensation to reduce the loading at the input node of the TIA.

Positive feedback Miller capacitances (C_F1_ and C_F2_) are introduced to reduce the effective capacitances at the X (C_X_) and Y (C_Y_) nodes in Figure 8b, while slightly increasing the parasitic capacitances at the Z (C_Z_) and O (C_O_) nodes, as described in (9) to (12). Here, A_1_, A_2_, and A_3_ denote the gains of each amplifier stage, and C_TX_, C_TY_, C_TZ_, and C_TO_ represent the parasitic capacitances introduced by the transistors at each node. On one hand, the effective capacitance at the X node is C_T_, as given by (7) and (8), which allows for a larger R_F_ while simultaneously enhancing the TIA bandwidth. This improvement also suppresses the third term of the input-referred noise current (f^2^ noise), as depicted in Figure 7. On the other hand, this structure allows for a larger input transistor size, thereby enhancing the g_m_, which reduces both the second and third terms of the input-referred noise current (thermal noise of input transistors and f^2^ noise, respectively). The associated increase in parasitic capacitance at the X and Y nodes is effectively compsensated by positive feedback Miller capacitance. Consequently, the optimal configuration is achieved, as illustrated in Figure 7b.

(9) $$C_{X}=C_{pad}+C_{PD}+C_{TX}+\left(1-A_{1}A_{2}\right)C_{F1}$$

(10) $$C_{Y}=C_{TY}+\left(1-A_{2}A_{3}\right)C_{F2}$$

(11) $$C_{Z}=C_{TZ}+C_{F1}/\left(1-1/A_{1}A_{2}\right)$$

(12) $$\;C_{O}=C_{TO}+C_{F2}/\left(1-1/A_{2}A_{3}\right)$$

**Figure 9:**
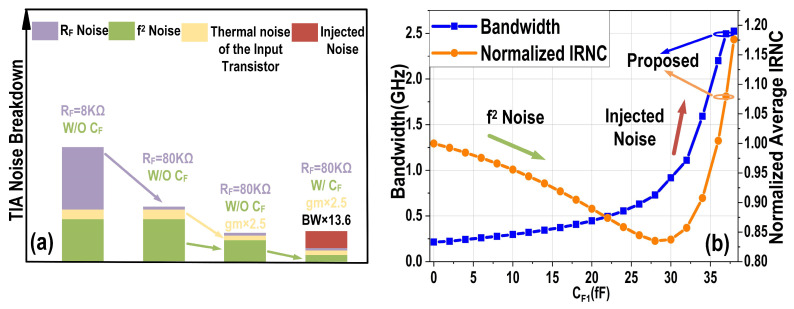
(**a**) Comparison of the TIA noise breakdown; (**b**) balanced homodyne receiver bandwidth and normalized input-referred noise current (IRNC) with different C_F1_ values.

Figure 9a provides a visual comparison of the TIA’s noise breakdown for various R_F_, g_m_, and C_T_ configurations, clearly illustrating a significant reduction in all components of the TIA’s input-referred noise current. Here, C_PD_ + C_pad_ = 300 fF represents the combined parasitic capacitance of two PDs and three PADs. However, increasing C_F1_ does not result in a monotonically decreasing input-referred noise current, as it also introduces the noise voltage from the output of the second amplifier stage back into the TIA input. Figure 9b depicts the impact of C_F1_ on the TIA’s bandwidth and noise performance. While a larger C_F1_ consistently increases the bandwidth, it simultaneously injects additional noise into the input. This injected noise gradually offsets the noise reduction benefits originally provided by the positive feedback Miller capacitors, thereby limiting the overall improvement in input-referred noise current.

Nevertheless, the use of positive feedback Miller capacitance severely degrades the phase margin of the TIA loop. The phase margin decreases from 82° to 25° when there is no inductive peaking at the Z and O nodes. By adjusting the values of the inductors and the size of the cascode transistors, the poles at Y and Z nodes are pushed to higher frequencies, restoring the phase margin of the TIA loop to 67.5°. The phase margin is particularly sensitive to C_F1_ because node X serves as the main pole of the TIA loop, directly affecting the loop’s phase margin, therefore a slight reduction in C_F1_ is necessary to ensure the loop’s stability. The TIA is followed by a single-to-differential (S2D) converter with a g_m_/g_m_ structure, ensuring very low total harmonic distortion (THD) [15]. The signal is then delivered via a buffer stage, as shown in Figure 8c.

**Figure 10:**
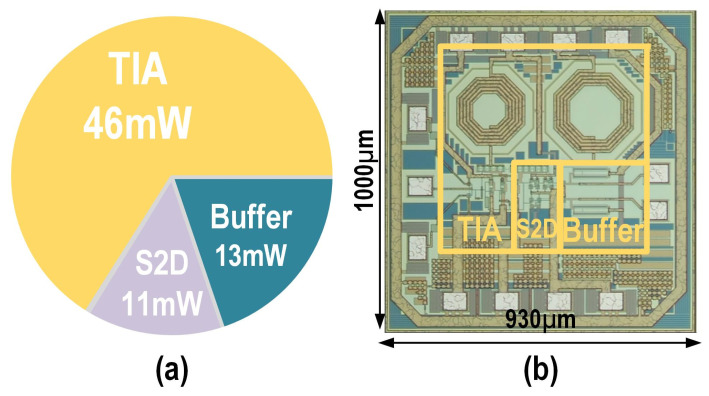
(**a**) Power consumption breakdown and (**b**) die micrograph of the EIC.

Through optoelectronic co-design, the proposed balanced homodyne receiver achieves noise optimization while delivering high bandwidth, effectively overcoming the limitations imposed by BHD’s parasitic capacitance on EIC performance. Additionally, a carefully designed feedback loop ensures system stability by providing a sufficient phase margin. Figure 10a illustrates the power consumption breakdown of the proposed EIC. At a supply voltage of 2 V, the total power consumption is 70 mW, with the majority consumed by the low-noise TIA. Figure 10b shows the die micrograph of the fabricated EIC using a 180 nm CMOS process, which is 1000 μm in height and 930 μm in width.

## 4. Measurement Results

**Figure 11:**
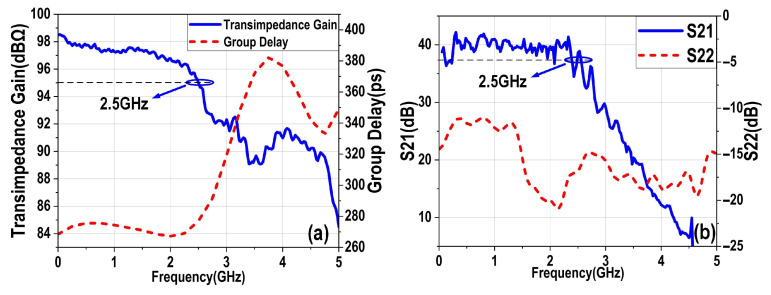
(**a**) Measured transimpedance gain and group delay. (**b**) Measured S21 and S22 parameters of the proposed EIC.

We first evaluated the proposed EIC to verify its bandwidth and noise performance. Figure 11a,b present the measured electronic performance of the proposed EIC, tested with 300 fF capacitance at input to match the load condition of the optoelectronic co-design. The EIC achieves a transimpedance gain of 98 dBΩ, a 3 dB bandwidth of 2.5 GHz, and an S_22_ below −10 dB. The measurement results confirm that the proposed EIC meets the design specifications, demonstrating the feasibility and effectiveness of the circuit architecture.

**Figure 12:**
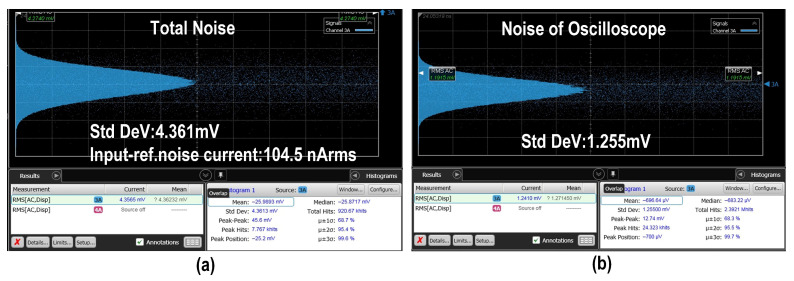
Measured RMS noise of (**a**) the EIC output and (**b**) the oscilloscope.

**Figure 13:**
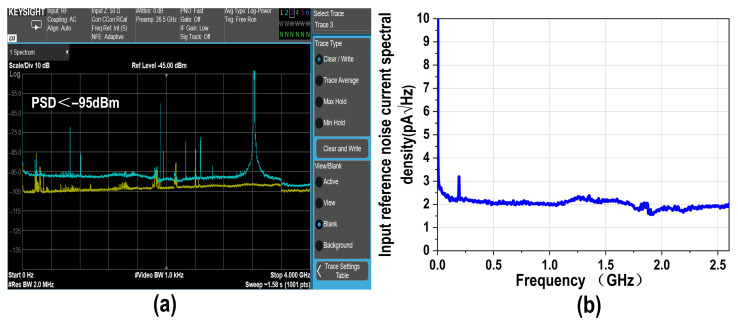
(**a**) Measured output noise PSD of the EIC and (**b**) input-referred noise current spectral density of the proposed EIC.

Subsequently, we conducted noise performance measurements on the proposed balanced homodyne receiver. The integrated noise results of the EIC output and the oscilloscope noise are shown in Figure 12a,b, yielding an input-referred noise current of 104.5 nA rms, calculated by dividing the amplitude of the output noise (Std Dev) by the transimpedance gain of the TIA. Figure 13a illustrates the measured noise power spectral density (PSD), remaining below −95 dBm within the 0–4 GHz range. The corresponding input-referred noise current derived from the PSD is shown in Figure 13b, obtained by dividing the PSD by the square root resolution bandwidth (√RBW) and then by the transimpedance gain. The average input-referred noise current of the proposed TIA is approximately 2 pA/√Hz.

**Figure 14:**
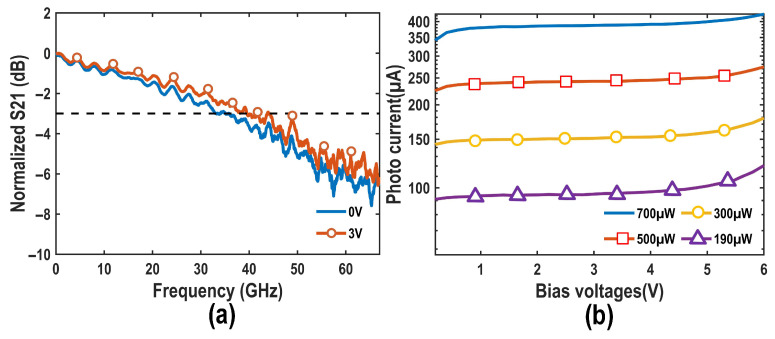
Measured optical characteristics of the proposed PD. (**a**) S21; (**b**) Photocurrent.

Figure 14a,b illustrate the measured bandwidth of the PD under varying bias voltages and the relationship between output photocurrent and input optical power. The PD achieves a 3 dB bandwidth of 40 GHz at a bias voltage of 3 V, with a maximum output photocurrent of approximately 350 μA when the input optical power is 700 μW. Subsequently, measurements of the quantum shot noise clearance were conducted. After the optical signal input, the photocurrent offset of the two PDs was determined by measuring the DC voltage output from the EIC, and the symmetry of the optical path was adjusted using a phase shifter, restoring the output DC offset from 1.317 V to 1.302 V.

**Figure 15:**
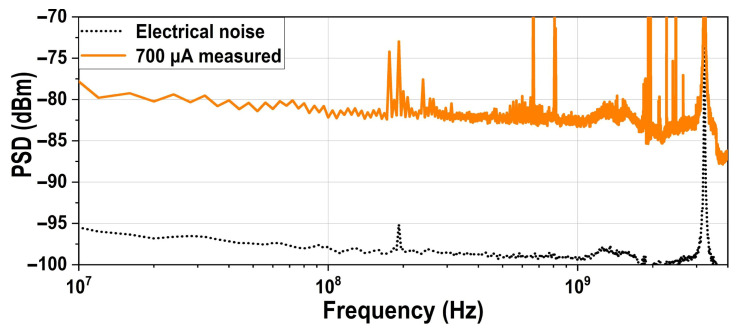
Measured noise PSD at the output of the proposed balanced homodyne receiver with and without the shot noise generated by a 700 μA photocurrent.

Figure 15 compares the PSD with and without quantum shot noise generated from total photocurrent of 700 μA, revealing a 15 dB increase in noise power. According to (13), with a total input photocurrent of 1 mA, $I_{AT}$ and $I_{AB}$ increase by a factor of 1.42 compared to 700 μA and the quantum shot noise clearance rises by 1.5 dB to reach 16.5 dB, where $I_{n,shot}^{2}$ is the shot noise density and $I_{AT}$ and $I_{AB}$ are the average currents flowing through the top and bottom PDs, respectively.

$$\begin{array}{cc} \mathrm{Clearance}\;= & 10{\;\log}_{10}(\frac{I_{n,shot}^{2}}{I_{n,TIA}^{2}})\;\mathrm{dB}\\ = & 10{\;\log}_{10}(\frac{2qI_{AT}+2qI_{AB}}{I_{n,TIA}^{2}})\;\mathrm{dB}\; \end{array}$$

**Figure 16:**
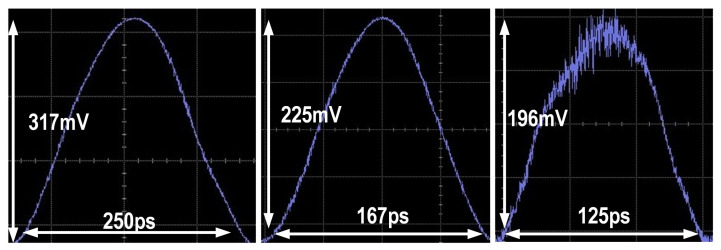
Pulse sampling performance of the proposed balanced homodyne receiver with pulse widths of 125 ps, 167 ps, and 250 ps.

**Figure 17:**
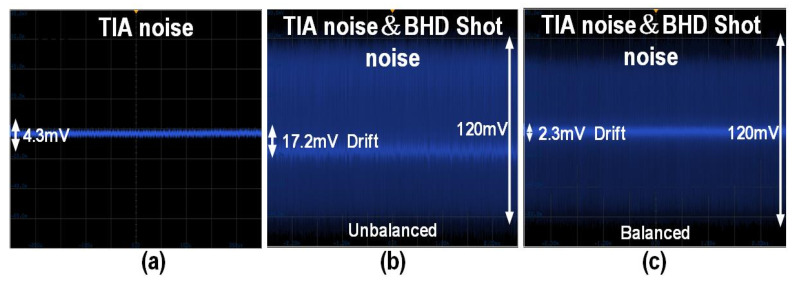
(**a**) Time-domain comparison of TIA noise only; (**b**) the combined quantum shot noise and TIA noise of the proposed balanced homodyne receiver without phase adjustment; and (**c**) the combined quantum shot noise and TIA noise of the proposed balanced homodyne receiver with phase adjustment.

The proposed balanced homodyne receiver’s ability to capture quantum fluctuations is demonstrated in Figure 16, showing its capability to clearly sample pulse signals with a width of only 125 ps. Figure 17a–c visually compare the electronic noise of the TIA with the shot noise of the BHD system and the process of adjusting the receiver’s symmetry using the proposed phase shifter. This highlights the proposed balanced homodyne receiver’s ability to effectively amplify quantum information and the effectiveness of the proposed adjustment scheme for optical path balance. Ultimately, the proposed solution reduces the 17.2 mV DC drift caused by optical path asymmetry to 2.3 mV. Compared to the 120 mV output amplitude of shot noise, the common-mode rejection ratio (CMRR) of the proposed balanced homodyne receiver improves from 16.8 dB to 34.3 dB.

**Figure 18:**
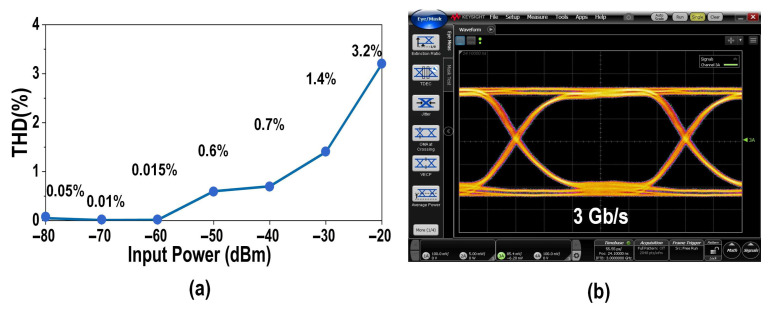
(**a**) Measured THD at different input power levels; (**b**) Measured eye diagram at 3 Gb/s.

To verify the stability of the proposed balanced homodyne receiver, noise performance validation was conducted in an environment without a shielded enclosure. Measurement results indicate that PSD at only partial frequency points undergoes random sudden changes introduced by environmental noise, with the measurement duration lasting one hour. Simultaneously, the THD of the EIC was measured, as shown in Figure 18a. At power inputs below −20 dBm, the TIA can achieve a THD of less than 3.2%. Finally, the phase margin of the TIA loop using the transient response (without shielding box) is verified, as described in Figure 18b. The EIC produced a excellent clear eye diagram with no overshoot at 3 Gb/s with PRBS31 input data, demonstrating robust signal integrity and TIA loop stability.

**Figure 19:**
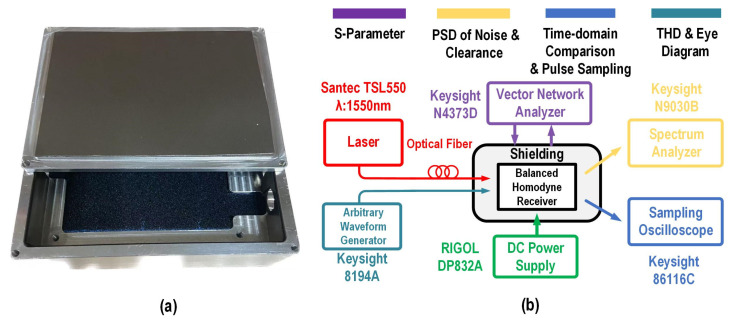
(**a**) Physical diagram of the shielding box. (**b**) Measurement setup of the proposed balanced homodyne receiver.

Figure 19a shows the physical diagram of the aluminum shielding box used in this work, with dimensions of 12 cm in length, 10 cm in width, and 6 cm in height. Figure 19b depicts the measurement setup for the proposed balanced homodyne receiver, where the S-parameters of the EIC are measured by a vector network analyzer, model Keysight N4373D. The noise PSD of the EIC and the quantum shot noise clearance of the proposed balanced receiver were measured using a spectrum analyzer, model Keysight N9030B. Using a Keysight 86116C sampling oscilloscope, the receiver’s pulse sampling capability was verified, and a visual comparison of shot noise and electronic noise in the time domain was conducted. THD and eye diagram measurements were performed by generating input signals using an arbitrary waveform generator, model Keysight 8194A, with output results measured using a spectrum analyzer and a sampling oscilloscope, respectively. The DC power supply module is RIGOL DP832A, providing a 2 V voltage, and the laser source emitting at a wavelength of 1550 nm is Santec TSL550.

A performance summary and comparison with state-of-the-art designs are provided in Table 2, highlighting the competitive noise performance and the maximum quantum shot noise clearance achieved with 1 mA photocurrent. To the best of our knowledge, the proposed balanced homodyne receiver exhibits the lowest input-referred noise current reported to date, along with the highest measured quantum shot noise clearance.

## 5. Discussion

The measurement results of the proposed balanced homodyne receiver align with design expectations, overcoming the trade-off between bandwidth and noise performance in TIA circuits. The adopted positive feedback Miller capacitor technique, combined with the BHD’s optoelectronic hybrid model, significantly reduces the input-referred noise current of the TIA while maintaining sufficient bandwidth, thereby optimizing the receiver’s sensitivity. Simultaneously, the phase shifter was employed to optimize the balance of the BHD link. As evidenced by the transient measurement results of the shot noise, the outputs from both photodetectors exhibit high symmetry.

The proposed balanced homodyne receiver significantly enhances the sensitivity of CV-QKD systems, which is crucial for quantum information measurements. Compared to reported TIA for CV-QKD systems, despite employing a relatively outdated CMOS process technology, the proposed TIA achieves the lowest input-referred noise current known to date for above GHz bandwidth. The scheme introduced in this work to reduce the input-referred noise current is instructive.

However, the THD performance of the proposed balanced homodyne receiver is constrained by the high gain of the EIC (RF = 80 kΩ), exhibiting THD below 3.2% only when the input electrical power is less than −20 dBm, and the saturation current of a single PD is only 350 μA. This reduces the LO’s amplification effect on the signal light, as the maximum optical signal the receiver can accept is limited. Once the signal becomes too large, the EIC’s output will ultimately become severely distorted. Meanwhile, adjusting the optical path balance using the thermo-optic effect of the phase shifter is susceptible to temperature, environmental, LO phase noise or drift, and manufacturing variations. In this work, phase shifter adjustments were manually controlled based on EIC output. To enhance system integrity and improve CMRR, a common-mode feedback loop will be incorporated in subsequent work, enabling automatic internal regulation of optical path balance performance.

Additionally, connecting the two PDs to the TIA input terminals via two bond wires reduces the parasitic inductance of the interconnect while also decoupling the parasitic capacitance between the two PDs, further enhancing the receiver’s bandwidth [18]. This approach can be employed to mitigate the sensitivity of hybrid-integrated BHD systems to bonding wire parasitics, thereby improving system stability.

The balanced design for PICs introduced in this work can be applied in coherent detection and quantum measurement fields. The proposed TIA structure can be widely used in analog front ends requiring high sensitivity and low noise performance. We believe that future research in this field will adopt more advanced integrated processes to achieve balanced homodyne receivers with even higher bandwidths and lower noise levels.

## 6. Conclusions

An adjustable integrated optical path is introduced to enhance the balance within the BHD system. By employing an equivalent circuit model of the PD and conducting optoelectronic co-design, a low-noise hybrid-integrated balanced homodyne receiver is demonstrated, fabricated in a 180 nm CMOS process for continuous-variable quantum information systems. The proposed TIA achieves a transimpedance gain of 98 dBΩ, a bandwidth of 2.5 GHz, and an input-referred noise current as low as 2 pA/√Hz, effectively overcoming the limitations imposed by BHD’s parasitics on bandwidth and noise performance. The balanced homodyne receiver achieves a quantum shot noise clearance of 15 dB with an input photocurrent of only 700 μA. Additionally, time-domain measurements provide an intuitive demonstration of the balanced homodyne receiver’s ability to capture quantum fluctuations, comparing the transient performance of shot noise and electronic noise.

## Figures and Tables

**Table 1:** Comparison of IM/DD and BHD.

	IM/DD	BHD
Bandwidth requirements	**High** (Should satisfy the Nyquist frequency)	**Mid** (I/Q modulation halaves the baud rate, ≤1/2 Nyquist frequency)
PD sensitivity requirements	**High** (Input optical signal determines the output amplitude)	**Mid** (Optical power of the input signal amplified by local oscillator)
Power consumption	**Low** (Direct drive & direct reception)	**High** (Multi-channel drive & more PIC bias, power consumption × 2)
Design complexity	**Low** (Simple structure & easy implementation)	**High** (Complex design & requires balance consideration)

**Table 2:** Performance summary and comparison.

Parameter	SCIENCEADVANCES 24 [1]	OPTICA 21 [2]	TCAS-I 19 [9]	TCAS-II 23 [16]	TCAS-II 24 [17]	This Work
Technology	250 nm Bi-CMOS	100 nm pHEMT	180 nm CMOS	180 nm CMOS	180 nm CMOS	**180 nm CMOS**
Integration method	Monolithic	Hybrid	Hybrid	Hybrid	Hybrid	**Hybrid**
Transimpedance gain	N/A	79 dBΩ	68.3 dBΩ	100 dBΩ	102 dBΩ	**98 dBΩ**
RF	600 Ω	N/A	3.5 kΩ	50 kΩ	18 kΩ	**80 kΩ**
BW_3dB_	15.3 GHz	1.5 GHz	8.5 GHz	260 MHz	151 MHz	**2.5 GHz**
BW_shot noise_/BW_PD_	N/A	40 GHz	N/A	N/A	N/A	**35 GHz**
Input-referred noise current	N/A	N/A	11.6 pA/√Hz	3.3 pA/√Hz	3.15 pA/√Hz	**2 pA/√Hz**
Clearance (From 1 mA photocurrent)	8 dB	12 dB	N/A	N/A	N/A	**16.5 dB**
Power consumption	N/A	850 mW	81 mW	153 mW	140 mW	**70 mW**
Die area of EIC (mm^2^)	0.01	5.76	0.78	0.89	0.741	**0.93**

## Data Availability

Data underlying the results presented in this paper are not publicly available at this time but may be obtained from the authors upon reasonable request.
